# Descenso en la esperanza de vida debido a los homicidios múltiples en México: homicidios de mujeres adyacentes al homicidio de uno o más hombres

**DOI:** 10.18294/sc.2023.4247

**Published:** 2023-03-23

**Authors:** Rosalba Jasso Vargas

**Affiliations:** 1 Doctora en Estudios de Población. Investigadora asociada C, Centro de Investigaciones Multidisciplinarias sobre Chiapas y la Frontera Sur, Universidad Nacional Autónoma México, Chiapas, México. rjasso@colmex.mx Universidad Nacional Autónoma México Centro de Investigaciones Multidisciplinarias sobre Chiapas y la Frontera Sur Universidad Nacional Autónoma México Chiapas México rjasso@colmex.mx

**Keywords:** Homicidio, Esperanza de Vida, Femicidio, México, Homicide, Life Expectancy, Femicide, Mexico

## Abstract

El objetivo es analizar el efecto de los homicidios múltiples sobre la esperanza de vida para la población masculina y femenina y ofrecer algunas evidencias empíricas sobre la correspondencia temporal y espacial entre las tasas de homicidio de hombres y mujeres, según grupo de edad, durante el periodo 2002-2020. A partir de datos del Instituto Nacional de Estadística y Geografía (INEGI) y de las proyecciones de población del Consejo Nacional de Población (CONAPO), se estimaron las tasas de mortalidad por homicidios en hombres y mujeres entre 2002 y 2020, se analizó la adyacencia de homicidios de hombres y mujeres, la relación espacial entre homicidios múltiples de hombres y mujeres y los cambios en la esperanza de vida debida a homicidios. Los homicidios unipersonales han provocado la mayor pérdida de esperanza de vida de hombres y mujeres. El efecto negativo sobre la esperanza de vida femenina y masculina debida a homicidios múltiples comienza a ser visible a partir de 2008. La exploración de los homicidios de mujeres adyacentes al homicidio de uno o más hombres plantea la inquietud de si estos crímenes son un resultado preponderante de la violencia delincuencial y en menor medida por razones de género.

## Abstract (en)

The objective is to analyze the effect of multiple homicides on male and female life expectancy and to provide empirical evidence of the temporal and spatial associations between male and female homicide rates by age group during the period 2002-2020. Using data from the National Institute of Statistics and Geography (INEGI) and population projections from the National Population Council (CONAPO), mortality rates due to homicide from 2002-2020 were estimated for both men and women, and the adjacency of homicides of men and women was analyzed, along with the spatial proximity between multiple homicides of men and women, along with changes in life expectancy due to homicide. Individual homicides have caused the greatest decrease in life expectancy for both men and women. The negative impact of multiple homicides on both female and male life expectancy began to draw attention in 2008. By analyzing the murders of women adjacent to the homicides of one or more men questions are raised about whether these crimes are primarily the result of criminal violence, and to a lesser extent the role of gender.

## INTRODUCCIÓN

Después de implementada la estrategia del gobierno mexicano de lucha frontal contra el crimen organizado a partir de 2007, que algunos han nombrado como “guerra contra las drogas”^1^, México ha sido escenario de constantes enfrentamientos entre las fuerzas gubernamentales y grupos criminales. Esta “guerra contra las drogas” refiere al cambio de estrategias desplegadas por el gobierno para hacer cumplir las políticas de drogas, cuya transformación más notoria ha sido el despliegue de soldados y marinos en tareas de seguridad pública para hacer frente a la delincuencia organizada^1^. A su vez, los ajustes, reacomodos y pugnas al interior de los grupos criminales son condiciones que se han traducido en un mayor número de homicidios^2^ Mientras México registró 10 homicidios por 100.000 habitantes a principio de siglo, una vez iniciada esta guerra, la tasa comienza a ascender hasta situarla, en 2018, en 29 homicidios por cada 100.000 habitantes^2^.

Si bien la magnitud de homicidios en el país es particularmente grave para la población masculina, el incremento de homicidios de mujeres es cada vez más alarmante. La tasa de homicidios de mujeres en 2019 ha sido la más alta registrada desde 1985, una tasa de 5,93 por cada 100.000 habitantes^3^ En términos de esperanza de vida, algunos autores han destacado la ralentización del incremento de la esperanza de vida de hombres y mujeres, principalmente para los primeros, para quienes incluso ocurre un estancamiento o reducción^4^^,^^5^

Los homicidios de mujeres son resultado, por una parte, de la violencia por razones de género y, además, de la violencia delincuencial. Las muertes originadas por el primer tipo de violencia suelen clasificarse como femicidios. En la literatura encontramos posicionamientos que sostienen que la violencia criminal incrementa la posibilidad de violencia de género. Específicamente, se ha documentado cómo la vulnerabilidad de las mujeres a sufrir violencia de género se incrementa en contextos de crimen organizado y de alta militarización^6^^,^^7^^,^^8^^,^^9^ Los homicidios, entonces, podrían ser resultado de una interrelación entre ambos tipos de violencia. Dada esta interdependencia entre violencias, cuantificar la dimensión de los femicidios es una tarea demás compleja. A pesar de la dificultad, se requiere avanzar en propuestas metodológicas que permitan una adecuada exploración de los homicidios de mujeres en contextos violentos.

La complejidad que entraña diferenciar entre femicidios y homicidios delincuenciales ha sido la principal motivación para explorar las muertes de mujeres en contextos de homicidios múltiples, es decir, que ocurren de manera adyacente al homicidio de uno o más hombres. La principal conjetura es que una importante proporción de homicidios de mujeres se encuentra vinculada a homicidios de hombres, y el homicidio de hombres suele ser ajeno a la premeditación para asesinarlas a ellas. La propuesta de este trabajo es que el homicidio de mujeres en este tipo de eventos se asociaría principalmente a razones criminales o delincuenciales y, en menor medida, a razones de género.

En este sentido, el objetivo del artículo es analizar el efecto de los homicidios múltiples sobre la esperanza de vida para la población masculina y femenina y ofrecer algunas evidencias empíricas sobre la correspondencia temporal y espacial entre las tasas de homicidio de hombres y mujeres, según grupo de edad, durante el periodo 2002-2020. Esta exploración empírica busca motivar la reflexión sobre los significados sociales de los homicidios múltiples en hombres y mujeres y sobre posibles caminos hacia la delimitación de los femicidios.

Es importante aclarar que existe un trabajo previo titulado “El asesinato de mujeres en eventos de homicidios de hombres en México, 2002-2020”^10^ en el que se presentaron las primeras ideas que son base de este documento, cuyo análisis se limitó a explorar las tasas de asesinato de hombres y mujeres a una escala nacional.

## MATERIAL Y MÉTODOS

Se realizó un estudio epidemiológico descriptivo, que se propone analizar las tasas de homicidios múltiples y homicidios unipersonales de hombres y mujeres y su efecto sobre la esperanza de vida. Los homicidios fueron clasificados -según el número de defunciones ocurridas, el lugar y la fecha- en dos tipos de eventos: *homicidio múltiple* (tres o más homicidios producidos el mismo día y en la misma localidad, que supondrían un vínculo entre sí); y *homicidio unipersonal* (hasta dos homicidios producidos el mismo día y en la misma localidad, que no supondrían un vínculo entre sí). 

Se parte del supuesto de que la cercanía en tiempo y espacio implicaría la ocurrencia de un mismo evento. En este sentido, por *adyacencia* se entiende un homicidio que ocurre de manera contigua o próxima a otro homicidio, el mismo día y en la misma localidad. Esta noción se utiliza para analizar la proporción de homicidios de mujeres adyacentes a homicidios de hombres y la proporción de homicidios en hombres adyacentes a homicidios de mujeres.

En primer lugar, se delimitaron los eventos de homicidios múltiples utilizando tres variables de corte temporal (día, mes y año) y tres variables de corte espacial (entidad federativa, municipio, localidad). En la medida en que alguna de estas variables estuviera erróneamente registrada, este tipo de eventos no podrían ser distinguidos. Del total de homicidios que ocurrieron entre 2002 y 2020, no se logró identificar si el homicidio fue múltiple o unipersonal en el 10,38% de los casos. La proporción de homicidios de hombres en los que no se pudo identificar el tipo de evento fue del 10,46% y, de mujeres, del 9,32%. En los casos en los que no se logró identificar el tipo de homicidio, se tomó la decisión de clasificarlos como homicidios unipersonales. De esta manera, el efecto de los homicidios múltiples sobre la esperanza de vida será el escenario más conservador para este conjunto.

Para calcular las tasas según año de ocurrencia (2002-2020) se emplearon las defunciones del Instituto Nacional de Estadística y Geografía (INEGI) y la población a mitad de año de las proyecciones de población del Consejo Nacional de Población (CONAPO). El término homicidio refiere en este trabajo a los decesos clasificados como “Agresiones” bajo los códigos X85-Y09 de la Clasificación Estadística Internacional de Enfermedades y Problemas Relacionados con la Salud (CIE-10). Con el fin de controlar el efecto de la estructura por edad se utilizó el método de estandarización directa y se tomó como base la población mexicana de ambos sexos a mitad del año 2020. Se describe la distribución temporal de las tasas de homicidios.

Se analiza la relación entre el logaritmo natural de las tasas estandarizadas de homicidios múltiples de hombres y mujeres según entidad federativa y año de ocurrencia. Esta exploración permite identificar, por un lado, las tasas de homicidios múltiples en determinadas entidades federativas y periodos y, por otro, la relación entre las tasas de homicidios de mujeres y hombres. Se utiliza el lugar de ocurrencia en lugar de la residencia habitual, pues esta última toma mayor relevancia cuando se analizan las muertes por causas violentas^11^

En la construcción de las tablas de mortalidad por todas las causas de muerte, se obtuvieron las tasas de mortalidad según grupo de edad, sexo, entidad federativa y año de ocurrencia. En esta tarea se emplearon las defunciones y población a mitad de año de las proyecciones de población del CONAPO. El siguiente paso consistió en la estimación de la esperanza de vida excluyendo las defunciones por homicidios. Para este propósito se asumen los supuestos del *multiple decrement processes*^12^.

La pérdida de esperanza de vida debido a los homicidios se estima a partir de la diferencia entre la esperanza de vida al nacer, excluyendo los homicidios, y la esperanza de vida al nacer que considera todas las causas de muerte. La contribución al cambio de la esperanza de vida según el grupo de edad se basó en el algoritmo “*stepwise replacement”*^13^. Este método es una herramienta universal para la descomposición de la diferencia entre medidas agregadas como la esperanza de vida^14^ El código en R se construyó con base en el algoritmo desarrollado por Jdanov y Shkolnikov “*the contour replacement*”^15^ Se calcula también el cambio en la esperanza de vida a los 20 y 40 años con el fin de descartar variaciones en el patrón temporal debido al nivel de subregistro.

Esta investigación emplea las bases de mortalidad del INEGI^16^ y las proyecciones oficiales del país, elaboradas por el CONAPO^17^ La información de ambas instituciones es de acceso público y disponible para su descarga. La publicación de estas bases de datos se realiza en apego a la reserva de información personal, por lo que el uso de esta información no representa riesgo alguno para la población bajo estudio.

## RESULTADOS

El objetivo del artículo es analizar el efecto de los homicidios múltiples sobre la esperanza de vida para la población masculina y femenina y ofrecer algunas evidencias empíricas sobre la correspondencia temporal y espacial entre las tasas de homicidio de hombres y mujeres, según grupo de edad, durante el periodo 2002-2022. En primer lugar, se presentan las tasas de mortalidad por homicidios a nivel nacional para hombres y mujeres a lo largo del periodo de estudio. En segundo lugar, para comprender el vínculo entre los homicidios de hombres y mujeres, se analiza la proporción de homicidios de mujeres adyacentes a homicidios de hombres y de hombres adyacentes a mujeres. En tercer lugar, se analiza la relación entre las tasas de homicidio múltiple de mujeres y hombres según entidad federativa y año de ocurrencia. Por último, se analizan los cambios en la esperanza de vida en hombres y mujeres, para todas las causas y excluyendo los homicidios.

### Análisis temporal

**Figura 1:**
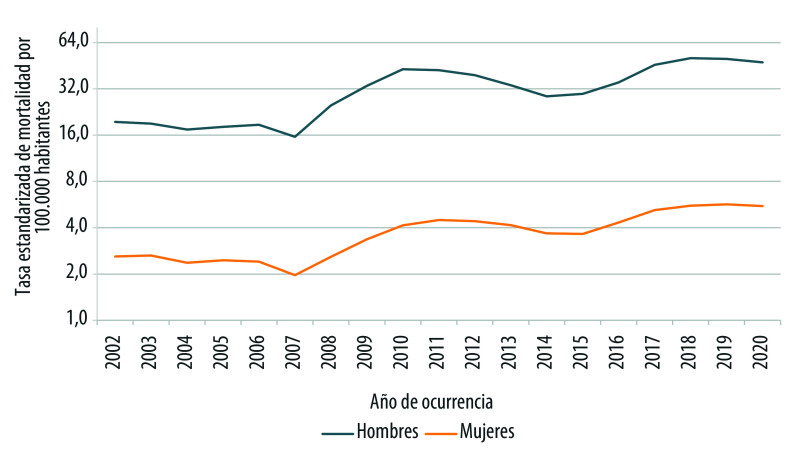
Tasas estandarizadas de homicidio de hombres y mujeres según año de ocurrencia (por 100.000 habitantes). México, 2002-2020.

El patrón temporal de las tasas de homicidios de hombres y mujeres en el tiempo guarda cierta relación, si las tasas de hombres ascienden (o descienden) las tasas de mujeres también experimentan un incremento (o descenso) en su propia escala (Figura 1). Ahora bien, la diferencia entre las defunciones masculinas y femeninas por cada 100.000 habitantes se acentúa a partir de 2008. Mientras, en 2002, la diferencia corresponde a 17 defunciones por cada 100.000 habitantes, en 2018, la distancia es de 45 defunciones por cada 100.000 habitantes.

Fuente: Elaboración propia con base en las estadísticas de mortalidad del Instituto Nacional de Estadística y Geografía, y la población a mitad de año 2002-2020 tomada de las proyecciones de población 1950-2050 del Consejo Nacional de Población. Nota: La tasa estandarizada de mortalidad está presentada en escala logarítmica.

Esta tendencia temporal ha sido reportada por otros autores y con base en ella han referido que el comportamiento de las tasas de asesinatos de mujeres en la vía pública y por armas de fuego sugieren un vínculo con el crimen organizado^3^ y que los homicidios de mujeres son un efecto colateral de la delincuencia y la militarización en el país^1^^,^^18^^,^^19^ Ante esta sugerencia que relaciona el patrón de ocurrencia de los homicidios de mujeres con las acciones del crimen organizado, una tarea pendiente ha sido lograr distinguir entre aquellos homicidios vinculados con la violencia delincuencial de aquellos que ocurrieron por razones de género.

### Análisis de adyacencia

Para avanzar en este sentido, se analiza la proporción de homicidios de mujeres adyacentes a homicidios de hombres. En la Tabla 1 se observa que, a partir de 2008, la proporción de homicidios de mujeres adyacentes al de algún hombre fue del 31,65% mientras que, en 2007, fue del 19,73%. En los puntos álgidos de violencia, como en 2010, esta proporción alcanzó el 48,41%. Es interesante analizar la contraparte de homicidios de hombres, pues la mayoría de las muertes se vincula en menor medida a los homicidios de mujeres. La proporción de homicidios de hombres adyacentes al homicidio de alguna mujer presentó un rango entre 2,97 en 2007 y 15,66 en 2010 (Tabla 1).

**Tabla 1:** Homicidios de mujeres en adyacencia de hombres y de hombres en adyacencia a mujeres, según año de ocurrencia. México, 2002-2020.

Año de ocurrencia	MUJERES				HOMBRES			Proporción HH adyacentes a HM
	HM sin HH	HM adyacente a HH	Total	Proporción de HM adyacentes a HH	HH sin HM	HH adyacente a HM	Total	
2002	833	187	1.020	18,33	7.203	228	7.431	3,07
2003	867	215	1.082	19,87	7.188	253	7.441	3,40
2004	819	158	977	16,17	6.716	195	6.911	2,82
2005	852	209	1.061	19,70	7.074	244	7.318	3,33
2006	847	196	1.043	18,79	7.527	240	7.767	3,09
2007	712	175	887	19,73	6.493	199	6.692	2,97
2008	827	383	1.210	31,65	10.610	895	11.505	7,78
2009	1.042	582	1.624	35,84	14.420	1.489	15.909	9,36
2010	1.118	1.049	2.167	48,41	18.127	3.366	21.493	15,66
2011	1.276	1.124	2.400	46,83	19.181	2.679	21.860	12,26
2012	1.357	963	2.320	41,51	17.818	1.953	19.771	9,88
2013	1.484	754	2.238	33,69	16.269	1.172	17.441	6,72
2014	1.374	611	1.985	30,78	14.210	852	15.062	5,66
2015	1.365	616	1.981	31,10	14.824	919	15.743	5,84
2016	1.602	858	2.460	34,88	18.098	1.393	19.491	7,15
2017	1.725	1.241	2.966	41,84	23.201	2.245	25.446	8,82
2018	1.722	1.526	3.248	46,98	24.925	3.233	28.158	11,48
2019	1.859	1.503	3.362	44,71	25.781	3.042	28.823	10,55
2020	1.777	1.630	3.407	47,84	25.078	3.326	28.404	11,71
Total	23.458	13.980	37.438	37,34	284.743	27.923	312.666	8,93

Fuente: Elaboración propia con base en las estadísticas vitales del INEGI. Nota: Se excluyeron los asesinatos que ocurrieron en eventos en los cuales no se identificó el sexo, la fecha o la entidad federativa de ocurrencia. Adyacencia= Homicidio que ocurre de manera contigua o próxima a otro homicidio, el mismo día y en la misma localidad. HM= Homicidio de mujeres. HH= Homicidio de hombres.

### Correspondencia espacial de los homicidios múltiples

Es importante destacar que el rango de las tasas de homicidios se incrementó en uno o dos dígitos a partir de 2008. Mientras que, entre 2002 y 2008, las tasas de homicidios de mujeres rondaban entre 0 y 8 por cada millón de mujeres, a partir de 2008, las tasas se ubicaron entre 0 y 273 por cada millón de mujeres. En los hombres, las tasas cambian de entre 0 y 80 a una amplitud de entre 0 y 1.500 homicidios por millón de hombres.

En la Tabla 2 se presenta el parámetro de la pendiente de la regresión lineal entre las tasas de homicidios de mujeres y hombres en homicidios múltiples según año de ocurrencia. Entre 2008 y 2020, la relación entre las tasas de homicidios múltiples para hombres y mujeres es lineal creciente, mientras que entre 2002 y 2007 el patrón se encuentra menos definido, incluso el coeficiente de determinación de los modelos lineales es menor que 0,76 antes de 2008. En general, después de 2008, la pendiente de la recta refleja que un incremento de una unidad en las tasas de homicidios múltiples de hombres corresponde a un incremento de 0,10 de la tasa de homicidio múltiples de mujeres.

**Tabla 2:** Parámetro de la pendiente de la regresión lineal entre las tasas de homicidios de mujeres y hombres en homicidios múltiples según año de ocurrencia. México, 2002-2020.

Año de ocurrencia	Pendiente	IC95%	Coeficiente de determinación del modelo
2002	0,078	0,050; 0,106	0,518
2003	0,193	0,150; 0,235	0,739
2004	0,071	0,036; 0,107	0,361
2005	0,096	0,050; 0,142	0,380
2006	0,108	0,086; 0,131	0,760
2007	0,097	0,031; 0,164	0,230
2008	0.066	0,063; 0,069	0,986
2009	0,064	0,057; 0,071	0,917
2010	0,105	0,099; 0,110	0,982
2011	0,109	0,099; 0,118	0,950
2012	0,101	0,088; 0,114	0,892
2013	0,093	0,079; 0,106	0,874
2014	0,110	0,089; 0,130	0,797
2015	0,093	0,077; 0,110	0,816
2016	0,101	0,090; 0,112	0,923
2017	0,115	0,103; 0,126	0,931
2018	0,107	0,100; 0,113	0,975
2019	0,118	0,112; 0,123	0,985
2020	0,127	0,119; 0,135	0,971

Fuente: Elaboración propia con base en las estadísticas vitales del Instituto Nacional de Estadística y Geografía. IC95%= Intervalo de confianza del 95%.

La entidad federativa de Chihuahua presenta las tasas más altas de homicidios para hombres y mujeres, principalmente, en 2010 y 2011. Quintana^20^ refiere que lo que se vive en Chihuahua (desde marzo 2008, con el Operativo Conjunto Chihuahua) no solo es violencia criminal, sino una violencia de Estado desplegada en las agresiones de diversos cuerpos policiacos y militares contra la población civil. De hecho, uno de cada tres homicidios de la guerra del presidente Calderón se ejecutó en este estado^20^ La entidad federativa que le sigue a Chihuahua es Baja California y luego Guerrero, exhibiendo una de las tasas de homicidios más altas, incluso previo a la explosión generalizada de violencia en todo el país. Específicamente, en el periodo 2013-2016, Guerrero presenta las tasas más altas en relación con el resto de las entidades federativas. En ningún año las tasas de homicidios de estas tres entidades federativas han sido 0, lo cual revela una violencia persistente en estos espacios. Otras entidades que se han caracterizado por altos niveles de violencia son Tamaulipas y Sinaloa. La violencia en estas entidades se manifiesta en menor medida en homicidios múltiples respecto a los primeros tres estados mencionados, pero superior al resto de las entidades federativas.

### El efecto de los homicidios sobre la esperanza de vida

Las tasas de homicidios más elevadas para hombres en relación con las de mujeres se traducen en una mayor pérdida de esperanza de vida, cuyo valor más alto ocurrió en 2018, con 1,23 años. En este mismo año, la pérdida de esperanza de vida para las mujeres fue de 0,17 años (2,07 meses). A pesar de las diferencias en las magnitudes de las tasas, siendo mayores entre los hombres respecto de las mujeres, vale la pena resaltar la similitud en los patrones de ocurrencia entre hombres y mujeres como acontecía con la distribución temporal de las tasas. Este patrón temporal se mantiene para la pérdida de esperanza de vida a los 20 y 40 años (Tabla 3 y Tabla 4).

**Tabla 3:** Pérdida de esperanza de vida al nacer, a los 20 y 40 años por homicidios en hombres, según año de ocurrencia, México, 2002-2020.

Año	Esperanza de vida al nacimiento			Esperanza de vida a los 20 años			Esperanza de vida a los 40 años		
	Todas las causas	Sin homicidio	Diferencia	Todas las causas	Sin homicidio	Diferencia	Todas las causas	Sin homicidio	Diferencia
2002	72,44	72,94	0,51	54,87	55,33	0,46	36,91	37,10	0,19
2003	72,48	72,94	0,50	54,80	55,26	0,46	36,83	37,03	0,20
2004	72,82	73,28	0,46	55,10	55,53	0,43	37,10	37,28	0,18
2005	72,66	73,15	0,48	54,90	55,35	0,44	36,95	37,13	0,18
2006	72,91	73,42	0,51	55,11	55,58	0,47	37,18	37,36	0,19
2007	72,72	73,14	0,43	54,88	55,28	0,40	37,03	37,19	0,15
2008	72,27	72,95	0,68	54,41	55,04	0,64	36,68	36,90	0,22
2009	71,91	72,79	0,88	54,01	54,84	0,82	36,39	36,67	0,28
2010	71,68	72,81	1,13	53,76	54,81	1,05	36,21	36,54	0,33
2011	71,87	73,01	1,14	53,91	54,96	1,05	36,37	36,70	0,33
2012	72,05	73,14	1,09	54,05	55,05	0,10	36,49	36,81	0,32
2013	72,15	73,11	0,96	54,09	54,98	0,89	36,50	36,80	0,30
2014	72,09	72,93	0,83	53,99	54,76	0,77	36,35	36,62	0,26
2015	71,88	72,74	0,86	53,74	54,53	0,80	36,07	36,35	0,28
2016	72,01	72,96	0,95	53,82	54,70	0,88	36,14	36,44	0,30
2017	72,05	73,21	1,16	53,85	54,92	1,07	36,16	36,52	0,36
2018	72,17	73,41	1,23	53,93	55,08	1,15	36,23	36,62	0,39
2019	72,27	73,47	1,20	54,00	55,11	1,12	36,27	36,65	0,37
2020	72,39	73,32	0,98	54,08	54,92	0,84	36,34	36,57	0,22

Fuente: Elaboración propia con base en las estadísticas de mortalidad del Instituto Nacional de Estadística y Geografía, y la población a mitad de año 2002-2020 tomada de las proyecciones de población 1950-2050 del Consejo Nacional de Población.

**Tabla 4:** Pérdida de esperanza de vida al nacer, a los 20 y 40 años por homicidios en mujeres, según año de ocurrencia, México, 2002-2020.

Año de ocurrencia	Esperanza de vida al nacimiento			Esperanza de vida a los 20 años			Esperanza de vida a los 40 años		
	Todas las causas	Sin homicidio	Diferencia	Todas las causas	Sin homicidio	Diferencia	Todas las causas	Sin homicidio	Diferencia
2002	77,77	77,85	0,08	59,75	59,81	0,06	40,52	40,55	0,03
2003	77,75	77,83	0,08	59,66	59,72	0,06	40,43	40,46	0,03
2004	78,05	78,12	0,07	59,91	59,96	0,05	40,66	40,69	0,02
2005	77,84	77,92	0,07	59,66	59,71	0,06	40,43	40,46	0,03
2006	78,23	78,31	0,07	60,01	60,06	0,06	40,77	40,80	0,03
2007	78,05	78,11	0,06	59,80	59,84	0,045	40,58	40,60	0,02
2008	78,10	78,18	0,08	59,81	59,88	0,06	40,60	40,63	0,03
2009	77,91	78,01	0,10	59,60	59,68	0,08	40,41	40,44	0,03
2010	77,93	78,06	0,13	59,59	59,69	0,10	40,40	40,44	0,04
2011	78,05	78,19	0,14	59,67	59,79	0,12	40,49	40,53	0,04
2012	78,05	78,19	0,14	59,64	59,75	0,11	40,46	40,50	0,04
2013	78,02	78,15	0,14	59,56	59,67	0,11	40,39	40,42	0,04
2014	77,87	77,99	0,12	59,38	59,48	0,10	40,21	40,25	0,04
2015	77,60	77,72	0,12	59,08	59,18	0,10	39,93	39,97	0,03
2016	77,73	77,87	0,14	59,18	59,29	0,11	40,02	40,06	0,04
2017	77,78	77,94	0,16	59,21	59,35	0,14	40,04	40,09	0,05
2018	77,91	78,08	0,17	59,31	59,45	0,14	40,12	40,17	0,05
2019	78,01	78,18	0,17	59,38	59,52	0,14	40,18	40,23	0,05
2020	78,14	78,27	0,13	59,48	59,58	0,11	40,27	40,30	0,03

Fuente: Elaboración propia con base en las estadísticas de mortalidad del Instituto Nacional de Estadística y Geografía, y la población a mitad de año 2002-2020 tomada de las proyecciones de población 1950-2050 del Consejo Nacional de Población.

**Figura 2:**
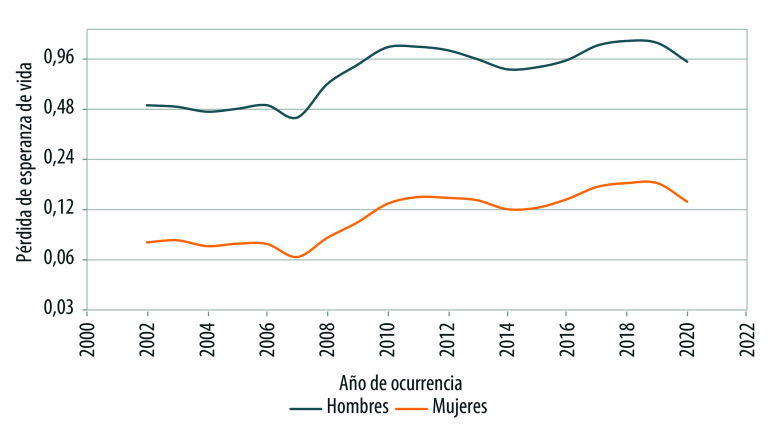
Pérdida de esperanza de vida por homicidios, según sexo y año de ocurrencia. México, 2002-2020.

En la Figura 1 y la Figura 2 se distinguen dos elevaciones: la primera centrada en 2010-2011 y la segunda en 2018-2019. A pesar de la similitud en el patrón temporal de las curvas, los periodos de mayor violencia afectaron de manera diferencial a hombres y mujeres. Mientras los dos puntos máximos para los hombres alcanzan una altura casi contigua, en el caso de las mujeres los pináculos se encuentran más distanciados, siendo el periodo de 2018-2019 el de mayor impacto.

Fuente: Elaboración propia con base en las estadísticas de mortalidad del Instituto Nacional de Estadística y Geografía, y la población a mitad de año 2002-2020 tomada de las proyecciones de población 1950-2050 del Consejo Nacional de Población. Nota: Los valores de la pérdida de esperanza de vida están presentadas en escala logarítmica.

Otro resultado de la exploración de la pérdida en la esperanza de vida debida a homicidios es que los incrementos y caídas son más pronunciadas para la población masculina que la femenina. Una explicación es que los cambios en la esperanza de vida de hombres son más volátiles en razón a una mortalidad más alta. Como lo explica Arriaga^21^ “los cambios relativos de las tasas específicas de mortalidad por edades producen incrementos de esperanza de vida más pequeños cuando el nivel de la mortalidad es bajo que cuando es alto”. En este sentido, previo al auge de violencia a partir de 2007, las mujeres gozaban de una esperanza de vida más alta (mortalidad más baja) respecto a los hombres y ante un escenario de mejoría cuando se excluyen hipotéticamente los homicidios, se producen cambios menores en la esperanza de vida de las mujeres.

Por lo que se refiere a los homicidios múltiples, a partir de 2008 estos eventos comenzaron a cobrar relevancia en determinados espacios. Así, por ejemplo, el año 2010 estuvo marcado por 237 acontecimientos que cobraron la vida de al menos 10 personas en conjunto; 91 eventos ocurrieron en 2011, 54 eventos en 2018 y 35 eventos en 2020. Espacialmente, en el periodo 2002-2020 han ocurrido 432 eventos en Chihuahua, 110 en Baja California, 27 en Guerrero, 25 en Tamaulipas, por mencionar los casos más trascendentes. Entonces, de los homicidios de más de 10 personas, el 62,32% ocurrió entre 2009 y 2011 y el 76,45% irrumpieron en Chihuahua y Baja California. Si bien los homicidios múltiples parecen concentrarse en ciertos espacios y episodios de violencia, en general, todas las entidades federativas mexicanas se han vuelto en promedio más violentas^19^ y se está experimentando un lamentable proceso de difusión^22^.

**Figura 3:**
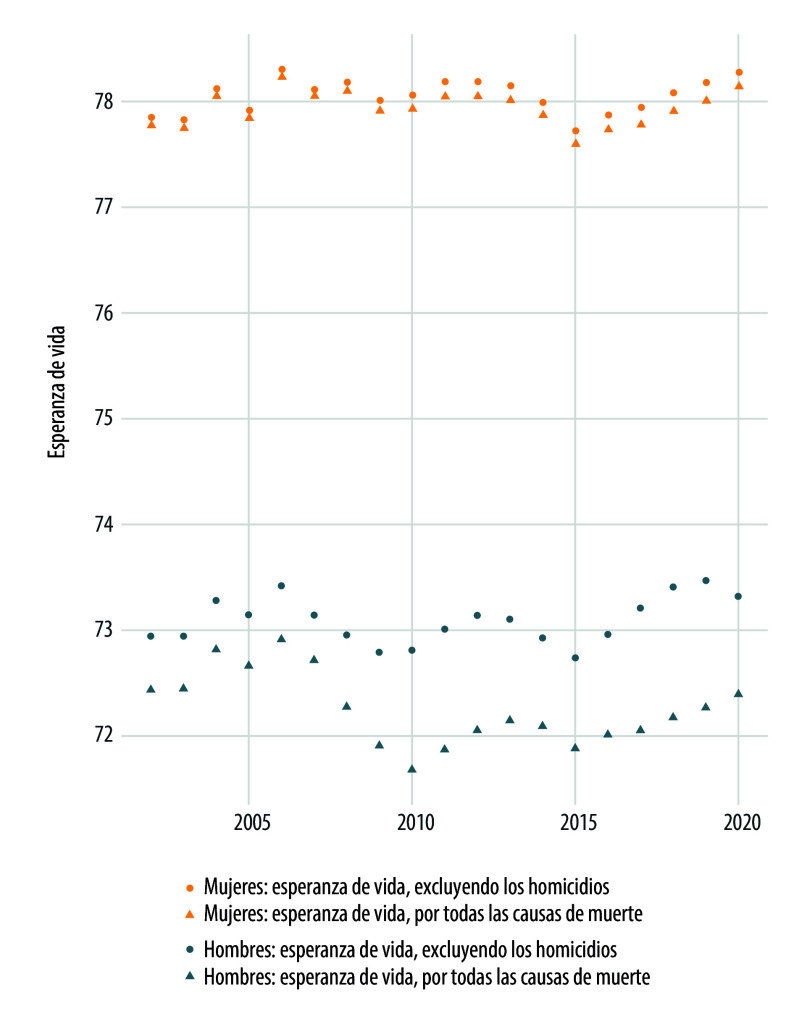
Esperanza de vida por todas las causas de muerte y excluyendo los homicidios según sexo y año de ocurrencia. México, 2002-2020.

En la Figura 3 se muestra la esperanza de vida considerando todas las causas de muerte y excluyendo los homicidios. Previo a 2007, la esperanza de vida de los hombres por todas las causas era cercana a los 73 años, después de este año comienza a descender, e incluso en 2010, se encuentra por debajo de los 72 años. Esta tendencia se ha mantenido y solo ha experimentado un ligero incremento en 2019 y 2020. Simultáneamente, la esperanza de vida de las mujeres ha oscilado alrededor de los 78 años sin exhibir un incremento como sería lo esperado. La explicación al estancamiento y descenso de la esperanza de vida en la década 2000-2010, identificada por otros autores, se debió principalmente a los homicidios entre las edades de 15 a 50 años y la enfermedad de diabetes mellitus a edades mayores a los 45 años^4^.

Fuente: Elaboración propia con base en las estadísticas de mortalidad del Instituto Nacional de Estadística y Geografía, y la población a mitad de año 2002-2020 tomada de las proyecciones de población 1950-2050 del Consejo Nacional de Población.

**Figura 4:**
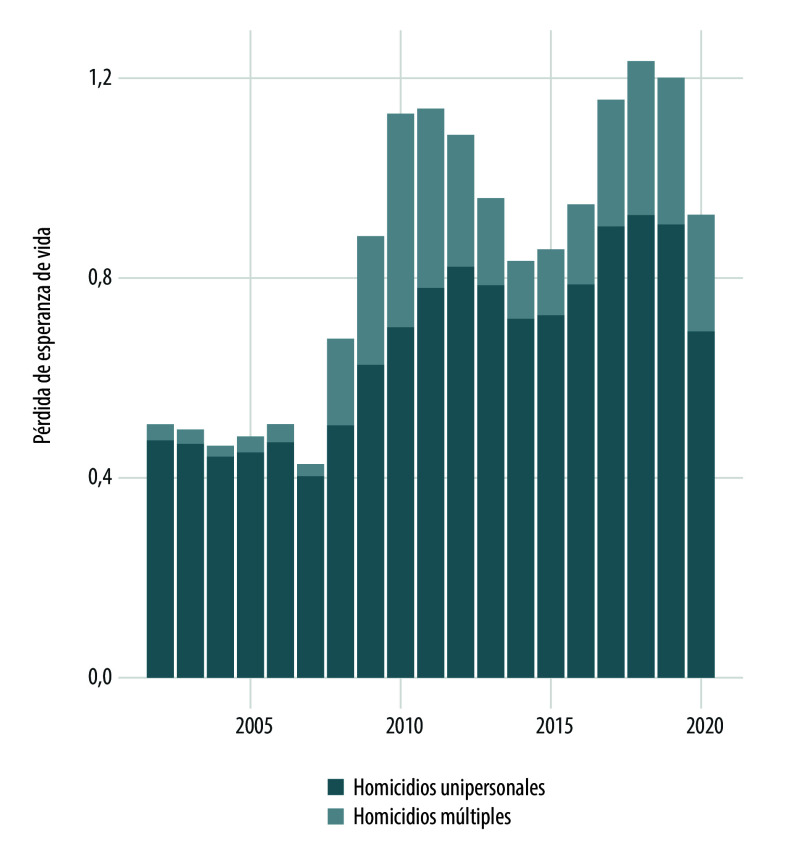
Pérdida de esperanza de vida en hombres debido a homicidios múltiples y unipersonales según año de ocurrencia. México, 2002-2020.

**Figura 5:**
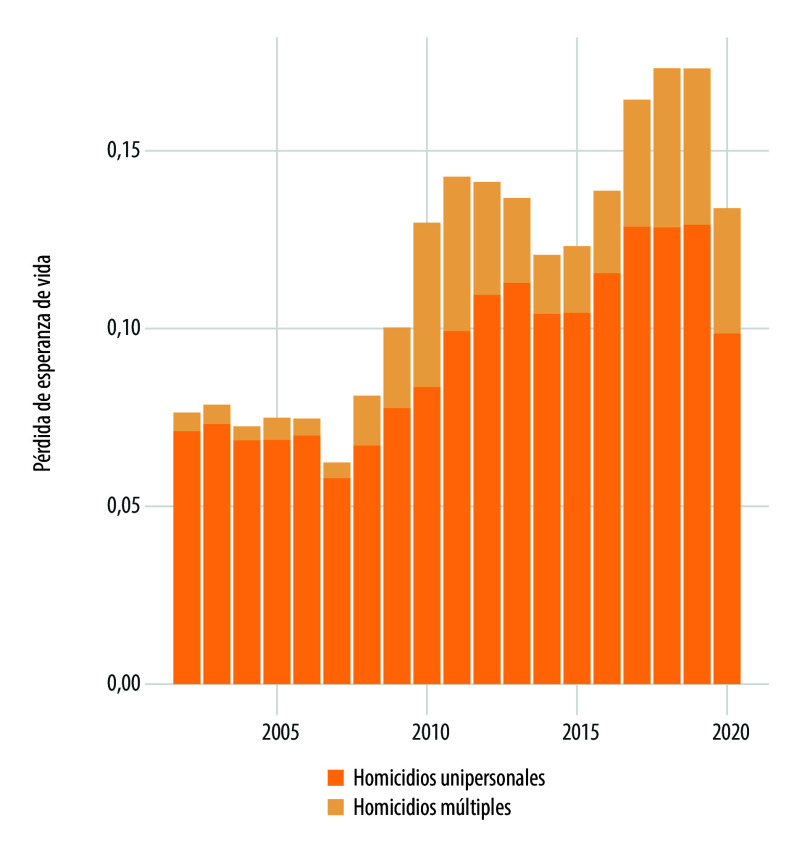
Pérdida de esperanza de vida en mujeres debido a homicidios múltiples y unipersonales según año de ocurrencia. México, 2002-2020.

Si se descomponen los cambios en la esperanza vida debido a los homicidios múltiples o unipersonales (Figura 4 y Figura 5), se observa que estos últimos eventos comienzan a mostrar su impacto más alto a partir de 2008 y alcanza sus niveles más altos en 2010-2011 y 2018-2019. Las mayores pérdidas en esperanza de vida se debieron principalmente a los homicidios unipersonales que, al igual que los homicidios múltiples, tomaron mayor relevancia a partir de 2008. Cabe subrayar que, previo a 2006, el efecto de los homicidios múltiples parecía permanecer constante y estable.

Fuente: Elaboración propia con base en las estadísticas de mortalidad del Instituto Nacional de Estadística y Geografía, y la población a mitad de año 2002-2020 tomada de las proyecciones de población 1950-2050 del Consejo Nacional de Población.

Fuente: Elaboración propia con base en las estadísticas de mortalidad del Instituto Nacional de Estadística y Geografía, y la población a mitad de año 2002-2020 tomada de las proyecciones de población 1950-2050 del Consejo Nacional de Población.

### Descomposición por grupos de edad

La contribución por grupos de edad presenta una preponderancia para los grupos centrales (entre 20 y 40 años) y este comportamiento es similar para los homicidios múltiples y unipersonales. El grupo de edad con la mayor contribución a la pérdida de esperanza de vida es el de 25 a 29 años para los hombres y el de 20 a 24 años para mujeres (con excepción del año 2015 para los homicidios unipersonales en mujeres). Los homicidios múltiples tienen su mayor impacto en 2010 y, principalmente, en 2018.

La contribución a la pérdida de esperanza de vida por homicidios unipersonales exhibe una mayor dispersión entre los grupos de edad. Las principales víctimas de los homicidios múltiples son jóvenes entre 12 y 50 años. Los homicidios de personas mayores a 60 años fueron, en su mayoría, unipersonales y parece responder a un fenómeno antiguo presente antes de los años más violentos del país (las curvas de homicidios unipersonales para los diferentes años se asemejan).

## DISCUSIÓN

La violencia desatada después del inicio de la “guerra contra las drogas”^1^ ha impactado en la vida de hombres y mujeres, cuyas secuelas se mantienen vigentes de manera alarmante. Las consecuencias han sido particularmente adversas en determinados periodos y estados de México, ocasionados en parte por el incremento de los homicidios múltiples.

El patrón temporal de las curvas de las tasas de homicidio para hombres y mujeres podría ser indicativa de que, como lo muestran otras investigaciones^1^^,^^3^, las ondas de violencia han afectado a ambos grupos poblacionales. Parece ser que los movimientos oscilatorios en las tasas de homicidios son generados en este contexto de lucha contra y entre los grupos criminales. Adicional a estas variaciones, existe una cuota desconocida de homicidios por razones de género (usualmente homicidios unipersonales) que a manera de conjetura se encuentran presentes previo a estas luchas.

El homicidio de mujeres debe pensarse desde al menos tres tipos de violencia: la violencia de género, la violencia delincuencial y una combinación de ambas. Debido a que los registros de muerte no permiten una adecuada delimitación de los femicidios, algunos autores han optado por analizar las defunciones con presunción de homicidio^1^^,^^3^^,^^19^^,^^23^^,^^24^^,^^25^. No obstante, la exploración de los homicidios de mujeres requiere un esfuerzo de diferenciación debido a que, si son resultado de fenómenos separados con mecanismos propios, las soluciones necesariamente tienen que ser diferenciales. Si bien este estudio no contribuye a una clara distinción operativa entre femicidio y homicidios de mujeres, intenta mostrar cómo un número significativo de homicidios de mujeres ocurren en eventos de homicidios múltiples, en los que algunos hombres estuvieron implicados y plantea la inquietud de si debiéramos excluir estas muertes en la delimitación de los femicidios.

En este sentido, este estudio planteó analizar los homicidios de hombres y mujeres en conjunto, y mostrar cómo los asesinatos entre ambos grupos se encuentran interrelacionados. De ahí que, una primera recomendación es examinar los homicidios de mujeres en relación con los homicidios de hombres. Este estudio explora las cifras de los homicidios múltiples y plantea la inquietud de si estos son consecuencia primordial de una violencia del tipo delincuencial y, en menor medida, por razones de género.

Un primer resultado es que cerca de la mitad de los homicidios de mujeres se encuentran vinculados a la muerte de algún hombre. A su vez, los homicidios de hombres se vinculan en menor medida a los homicidios de mujeres. Este resultado parece soportar la idea de que la violencia experimentada en el país es generada principalmente por hombres contra hombres y que algunas mujeres se han visto involucradas en eventos de violencia extensiva. De ahí el interés por analizar los homicidios múltiples en los que las mujeres se han visto implicadas.

Del desarrollo de este trabajo, cabe destacar dos resultados principales. El primero, la mayor pérdida de esperanza de vida de hombres y mujeres debido a los homicidios en el país ha sido originado por los homicidios unipersonales. El segundo, la existencia de un patrón diferenciado entre hombres y mujeres en edades jóvenes (20 a 40 años), que se expresa, por un lado, en que las mujeres, independientemente de los periodos de violencia, enfrentan un riesgo permanente y creciente de homicidio unipersonal (que se asocia con una parte de los femicidios); y, en forma simultánea, en los periodos de violencia enfrentan un mayor riesgo de homicidios unipersonales y, en menor medida, de los homicidios múltiples. Por otra parte, los hombres jóvenes, en periodos de violencia, experimentan mayor pérdida de esperanza de vida ocasionada por los homicidios múltiples y unipersonales.

Ahora bien, la contribución a la pérdida en la esperanza de vida en los grupos de edad mayores de 60 años es pequeña por dos razones (nula en el caso de homicidios múltiples). La primera, la dimensión de homicidios es menor en relación con los homicidios de personas en otros grupos de edad. La segunda, la contribución a la pérdida de la esperanza de vida en estos grupos de edad suele ser menor debido a que si se evitaran estos homicidios, la mejora en la esperanza de vida no tendría un efecto tan significativo como el que tendría reducir los homicidios en edades jóvenes. 

Otro elemento por destacar son las muertes de niñas en homicidios múltiples cuya pérdida en la esperanza de vida comienza a manifestarse después de iniciada la guerra contra el narcotráfico. Simultáneamente, el riesgo de muerte de los niños varones en este tipo de eventos también se incrementó durante los años de violencia, no obstante, sus efectos en la esperanza de vida no son claramente visibles debido a los impactos más altos para hombres jóvenes.

La pérdida de la esperanza de vida para las mujeres es diferencial en los dos periodos de mayor violencia (2010-2011 y 2018-2019) y la violencia experimentada en este último periodo ha sido más agresiva contra su prospectiva de vida. La diferencia entre ambos años se debe a la pérdida de la esperanza de vida ocasionada por los homicidios unipersonales en los grupos de edades centrales (20 a 40 años). Es decir, la contribución de los homicidios múltiples en ambos años (2010 y 2018) son próximas mientras la contribución de los homicidios unipersonales en 2018 es incluso cercana al doble, respecto a 2010. De hecho, las pérdidas provocadas por los homicidios unipersonales de mujeres en los grupos de edad jóvenes (20 a 40 años) son crecientes a través de los años. A diferencia de los homicidios de mujeres, los homicidios múltiples de hombres jóvenes (20 a 40 años) exhiben una contribución diferencial en estos años, siendo 2010 el año de mayor pérdida de la esperanza de vida. Aunque los hombres jóvenes también exhiben impactos negativos en su esperanza de vida debido a los homicidios unipersonales, en ciertos periodos parece mantenerse en cierto nivel. Por ejemplo, la pérdida en esperanza de vida para el grupo de edad de 25-29 años es de 0,1217 y 0,1233 en 2010 y 2015 respectivamente. Para el grupo de edad de 30-34 las pérdidas en estos mismos años son de 0,11 y 0,1118. En otras palabras, el impacto de los homicidios unipersonales para los hombres jóvenes puede contraerse en tiempos de menor violencia, no obstante, el impacto de los homicidios unipersonales para las mujeres parece mantenerse o incrementarse, pero difícilmente reducirse.

Estos resultados apuntan a la necesaria reflexión sobre los significados de los homicidios unipersonales para hombres y mujeres. En palabras de Atuesta y Vela^1^ los contextos y formas de homicidios para hombres y mujeres son diferenciados. Los homicidios unipersonales de las mujeres pueden simbolizar, por un lado, su mayor grado de vulnerabilidad en contexto de violencia criminal y, por otro, violencias de tipo intrafamiliar. En este sentido, aún queda mucho camino para lograr establecer si las crecientes cifras de femicidios son resultado de momentos de recrudecimiento de la violencia de tipo criminal como algunos sugieren^6^ o que incluso el femicidio se mantiene creciente aún en espacios considerados como no violentos.

Dejando de lado los homicidios en edades centrales, los homicidios de adultos mayores probablemente se encuentran vinculados sobre todo con una violencia del tipo familiar o doméstica. De ahí que, en la última década se hayan realizado diversos estudios que exploran la prevalencia de violencia intrafamiliar hacia las personas adultas mayores^26^. Por otra parte, los homicidios de niños pueden responder al menos a dos circunstancias: se ejerce mayor violencia o maltrato severo en la disciplina hacia los varones^27^ y al reclutamiento forzado de infantes para actividades delincuenciales. Sobre este último punto, se argumenta que la utilización de este grupo poblacional es redituable debido a que, al ser menor de edad puede evadir a las autoridades más fácilmente y en caso de ser detenidos, se les dota de asesoría jurídica gratuita y las sentencias tienen una duración menor a los cinco años^28^. Para los menores de un año, algunos autores suponen que se tratan de casos de homicidios por violencia intrafamiliar^27^ e incluso, alrededor de la mitad de los homicidios de menores de cinco años ocurren en el hogar^29^. En todo caso, el asesinato de niños y niñas es reprobable en todo sentido pues es una violencia ejercida desde la desigualdad de poder^30^.

Finalmente, existen tres principales limitaciones de la herramienta metodológica utilizada. La primera, la dimensión de homicidios en los cuales no se puede definir el tipo de eventos en que ocurrieron (variables con respuesta no especificada). La segunda, la elección de la variable espacial para determinar la coincidencia de eventos demográficos entraña un mayor o menor error de asignación a un mismo suceso. En este estudio se utiliza la localidad como la unidad espacial más pequeña. Como un elemento adicional de coincidencia podría emplearse el lugar donde ocurrió el homicidio (vivienda, escuela, oficina pública, calle, carretera, granja, etcétera), lamentablemente el nivel de no respuesta es alto, por lo que no puede ser incorporada como dimensión de análisis. La tercera, excluye eventos de violencia que están vinculados de forma temporal pero no espacial. Recientemente, en el país se han observado explosiones de violencia en diferentes estados durante el mismo día y que podrían formar parte de un mismo suceso delincuencial. Una recomendación sería incorporar en los certificados de defunción alguna variable que vincule uno o más muertes si pertenecieron a un mismo suceso.

El efecto de los homicidios sobre la esperanza de vida se ha estimado a escala nacional, no obstante, queda pendiente explorar lo que ocurre específicamente en las localidades urbanas (población mayor a 2.500 habitantes). La importancia de los entornos urbanos radica en que la mayoría de los homicidios han ocurrido en este contexto. Además, mientras aproximadamente el 69% de los homicidios ocurrían en localidades urbanas, con posterioridad a 2008, este porcentaje se incrementó en alrededor del 76%. De manera que los resultados aquí presentados podrían reflejar sobre todo lo que ocurre en el entorno urbano. Adicionalmente, la esperanza de vida al nacer en espacios urbanos suele ser mayor que en otros contextos, por lo que, el efecto de los homicidios en la esperanza de vida también es diferencial. 

En vías de ir perfeccionando las herramientas operativas para una adecuada delimitación de los femicidios, la propuesta de este estudio es que, en lugar de utilizar todas las defunciones con presunción de homicidios, deberían excluirse aquellos homicidios que ocurrieron en eventos de homicidios múltiples. El primer paso entonces es incorporar a nuestro análisis la coincidencia temporal y espacial de los eventos demográficos. Si se incorporan otras aristas de análisis a esta técnica metodológica estaremos ante la posibilidad de indagar empíricamente otras dimensiones aún no exploradas como el homicidio de mujeres adyacente al homicidio de sus hijos e hijas, en explosiones de violencia conectadas temporal o espacialmente, en espacios concebidos como no violentos donde prevalecen los homicidios unipersonales de las mujeres, entre otras.
